# Anti-inflammatory and Pro-apoptotic Effects of 18beta-Glycyrrhetinic Acid *In Vitro* and *In Vivo* Models of Rheumatoid Arthritis

**DOI:** 10.3389/fphar.2021.681525

**Published:** 2021-07-26

**Authors:** Yunhui Feng, Liyan Mei, Maojie Wang, Qingchun Huang, Runyue Huang

**Affiliations:** ^1^ College of Physical Education, Guangzhou University, Guangzhou, China; ^2^ The Second Affiliated Hospital of Guangzhou University of Chinese Medicine (Guangdong Provincial Hospital of Chinese Medicine), Guangzhou, China; ^3^ Center for Molecular Medicine, University Medical Center Utrecht, Utrecht, Netherlands; ^4^ Guangdong Provincial Key Laboratory of Clinical Research on Traditional Chinese Medicine Syndrome, Guangzhou, China; ^5^ State Key Laboratory of Dampness Syndrome of Chinese Medicine (The Second Affiliated Hospital of Guangzhou University of Chinese Medicine), Guangzhou, China; ^6^ Guangdong-Hong Kong-Macau Joint Lab on Chinese Medicine and Immune Disease Research, Guangzhou University of Chinese Medicine, Guangzhou, China

**Keywords:** 18beta-glycyrrhetinic acid, rheumatoid arthritis, inflammation, proliferation, forkhead box O3

## Abstract

18β-Glycyrrhetinic acid (18β-GA), an active component from *Glycyrrhiza glabra L.* root (licorice), has been demonstrated to be able to protect against inflammatory response and reduce methotrexate (MTX)-derived toxicity. This study was therefore designed to test the therapeutic possibility of 18β-GA on rheumatoid arthritis (RA) and to explore the underlying mechanism. LPS or TNF-α-induced inflammatory cell models and collagen-induced arthritis (CIA) animal models were applied in this study. Real-time quantitative PCR (RT-qPCR) was used to measure the mRNA levels of various cytokines and FOXO family members. The protein levels of molecules in the MAPK/NF-κB signaling pathway were analyzed using western blot. The cell proliferation assay and colony-forming assay were used to test the influence of 18β-GA on cell viability. The cell apoptosis assay and cell cycle assay were performed to detect the effect of 18β-GA on cell proliferative capacity by using flow cytometry. Hematoxylin and eosin (H&E) staining was performed to evaluate pathological changes after drug administration. The enzyme-linked immunosorbent assay (ELISA) was carried out for the detection of cytokines in serum. *In vitro*, we found that 18β-GA decreased the mRNA levels of IL-1β, IL-6, and COX-2 by inhibiting the MAPK/NF-κB signaling pathway in MH7A and RAW264.7 cell lines. Moreover, 18β-GA was able to suppress cell viability, trigger cell apoptosis, and G1 phase cell cycle arrest in our *in vitro* studies. 18β-GA dramatically enhanced the mRNA level of FOXO3 in both TNF-α- and LPS-induced inflammation models *in vitro*. Interestingly, after analyzing GEO datasets, we found that the FOXO3 gene was significantly decreased in the RA synovial tissue as compared to healthy donors in multiple microarray studies. *In vivo*, 18β-GA exhibited a promising therapeutic effect in a collagen-induced arthritis mouse model by alleviating joint pathological changes and declining serum levels of TNF-α, IL-1β, and IL-6. Finally, we observed that 18β-GA administration could mitigate liver damage caused by collagen or MTX. Collectively, the current study demonstrates for the first time that 18β-GA can inhibit inflammation and proliferation of synovial cells, and the underlying mechanism may be associated with its inhibition of MAPK/NF-κB signaling and promotion of FOXO3 signaling. Therefore, 18β-GA is expected to be a new drug candidate for RA therapy.

## Introduction

Rheumatoid arthritis (RA) is a common chronic autoimmune disease that affects 1% of the world’s population (Frisell et al., 2016). Unfortunately, by treating with current therapeutic strategies, not all patients can achieve low disease activity and clinical remission (Conigliaro et al., 2019). Moreover, drugs in treatment strategies, such as disease-modifying antirheumatic drugs (DMARDs) and nonsteroidal anti-inflammatory drugs (NSAIDs), are prone to serious side effects, such as infections, liver damage, and immune impairment in long-term use, thus significantly limiting their therapeutic efficacy (Klinkhoff, 2004; Majithia and Geraci, 2007; Smolen and Aletaha, 2015). Thereby, addressing lower-cost alternative treatments without such limitations and developing new interventions based on a better pathogenetic understanding are ongoing issues in RA management and research.

It is well known that both inflammatory infiltration and synovial hyperplasia are the key pathological features of RA. Inflammatory infiltration is characterized by the enrichment of different types of immune cells, such as macrophages, T cells, and other inflammatory cells in the lesional synovial tissue and articular cavity (Firestein, 2005; Mcinnes and Schett, 2011). After long-term exposure to the pro-inflammatory mediators, such as tumor necrosis factor alpha (TNF-α), interleukin 1β (IL-1β), and interleukin 6 (IL-6), synovial cells will produce a set of chemokines that recruit circulating immune cells to migrate to the affected tissue, thus further exacerbating and perpetuating joint inflammation (Brzustewicz and Bryl, 2015; Kosmaczewska et al., 2011). Meanwhile, in this inflammatory microenvironment, synoviocytes will shift to a hyperproliferative state, which will then amplify the inflammatory response (Mcinnes and Schett, 2011). Moreover, it has been demonstrated that the number and phenotype of macrophages will affect the development of disease (Huang et al., 2019). Therefore, targeting synoviocytes and immune cells such as macrophages to interrupt the vicious circle has emerged as a potential treatment strategy for RA.

18β-Glycyrrhetinic acid (18β-GA) is an active ingredient of *Glycyrrhiza glabra L.* root extract (licorice), which is concurrently used in many Chinese herbal formulas to play roles in reducing toxicity and synergistic effect. As a bioactive compound of licorice, 18β-GA shows potential inhibitory effects on cancer, inflammation, and microorganisms (Hung et al., 2017; Yang et al., 2017). Furthermore, 18β-GA is able to reduce chemical drug-induced hepatotoxicity and nephrotoxicity, such as etoposide (Cai et al., 2017), cisplatin (Ma et al., 2016), and methotrexate (MTX) (Mahmoud et al., 2017). These studies provide us with a hint for the development of 18β-GA as a new drug for RA therapy.

In the present study, we designed a series of *in vitro* and *in vivo* experiments to investigate the effects and mechanisms of 18β-GA in the treatment of RA. First, the results of *in vitro* studies showed that 18β-GA had promising anti-inflammatory effects on the reduction of various inflammatory cytokines (IL-6, IL-1β, and COX-2) through inhibition of the MAPK/NF-κB pathway. We also found that18β-GA suppressed cell proliferation by inducing cell apoptosis and G1 cell cycle arrest. In addition, 18β-GA elevated the mRNA level of forkhead box O3 (FOXO3), which is a transcription factor that likely functions as a trigger for apoptosis. Notably, we found that the expression of FOXO3 was down-regulated in the RA synovial tissue in the study of online microarray datasets. Not only that 18β-GA decreased the mRNA level of ki67, which is a well-known cell proliferation marker, but also when we tested the therapeutic potential of 18β-GA in a collagen-induced arthritis (CIA) animal model, 18β-GA alone and in combination with MTX significantly reduced the severity of arthritis as well as the serum levels of inflammatory mediators. Importantly, 18β-GA attenuates liver pathological injury caused by administration of MTX. Taken together, our results provide a basis for the development of 18β-GA as a potential drug for the treatment of RA.

## Materials and Methods

### Animals and House Conditions

DBA/1 mice (female, 20 ± 2 g) were obtained from Jiangsu Gem-Pharmatech Co., Ltd. (Jiangsu, China) and were housed in a specific pathogen-free (SPF) environment under standard conditions of 12 h light/dark cycle, 25°C, and 55 ± 5% humidity. The animal experiment was approved by the Ethics Committee of Laboratory Animals in Guangdong Provincial Hospital of Chinese Medicine. The animal handling procedures were carried out according to the principles of China’s legislation on animals use and care.

### Cell Culture

The immortalized human RA fibroblast-like synovial (FLS) cell line MH7A and the murine macrophage cell line RAW264.7 were purchased from Guangzhou Jenniobio Biotechnology Co., Ltd. (Guangzhou, China). Both cell lines were cultured in Dulbecco’s modified Eagle’s medium (DMEM) (Invitrogen Carlsbad, CA, United States) supplemented with 10% fetal bovine serum (FBS) (Invitrogen), 100 mg/ml streptomycin (Invitrogen), and 100 units/ml penicillin (Invitrogen) in an incubator containing 5% CO2. MH7A and RAW264.7 cells were seeded in a 6-well plate and cultured in FBS-free DMEM overnight before exposure to 1 μg/ml of LPS (cat. no. L2880; Sigma-Aldrich, Darmstadt, Germany) or 100 ng/ml of TNF-α (cat. no. 300-01A; Peprotech, United States) for 3 h. Cells were treated with different doses of 18β-GA (purity >97%; cat. no. 1507075; Aladdin Reagent Co., Ltd., Shanghai, China) along with the addition of LPS or TNF-α.

### Collagen-Induced Arthritis Mouse Model and Drug Administration

The collagen-induced arthritis (CIA) animal model, the most commonly used animal model of RA, was established in this study. Briefly, 2 mg/ml Bovine type II collagen and complete Freund’s adjuvant (Chondrex, Inc.) were emulsified and mixed in a 1:1 ratio before injecting into the foot and tail of mice for the induction of the arthritis model (100 μl per mouse). The booster immunization was carried out by administration of a mixture of incomplete Freud’s adjuvant (Chondrex, Inc.) and type II collagen following the same method of first immunization on day 21. One week after the booster immunization, the CIA mice were randomly divided into four groups of six mice each. Together with the blank control group, there were five groups in total: 1) control group (PBS-treated, each day, p.o.); 2) model group (PBS-treated, each day, p.o.); 3) 18β-GA group (45 mg/kg, each day, p.o.); 4) MTX group (2 mg/kg, three times a week, p.o.); and 5) 18β-GA combined with the MTX group (18β-GA 45 mg/kg, each day, p.o. and MTX 2 mg/kg, three times a week, p.o.).

### Hematoxylin and Eosin (H&E) Staining

Ankle tissues of mice were fixed in 10% formalin after dissociation and then decalcified using decalcification solution (a mixture of ethylenediaminetetraacetic acid, sodium tartrate, hydrochloric acid, and potassium tartrate) before embedding in paraffin. Liver tissues were fixed and embedded in paraffin directly. All samples were sliced into 3.5 -μm-thick sections and then used for hematoxylin and eosin (H&E) staining. The pathological score of joint sections was obtained by observing inflammatory cell infiltration, cartilage destruction and erosion, and synovial proliferation. Portal inflammation and hepatocyte morphology were applied to assess the extent of liver damage.

### Enzyme-Linked Immunosorbent Assay

On day 56, all mice were executed according to animal ethics guidelines. Blood samples were collected in coagulation-promoting tubes and then centrifuged at 3,000 rpm for 15 min to make serums. Mouse ELISA kits of TNF-α (Neobioscience, #EMC102a.96), IL-1β (Neobioscience, #EMC001 b.96), and IL- 6 (Neobioscience, #EMC004.96) were applied to measure the serum levels of relevant proinflammatory cytokines. All experimental operations were carried out according to manufacturer’s instructions. Absorbance was measured at 450 nm by using a termomax microplate reader (Bio-Tek, Winooski, United States).

### Cell Viability Assay

The effect of 18β-GA on cell viability was detected by using the CellTiter 96 Aqueous One Solution Cell Proliferation Assay (MTS) (Promega, Madison, United States) according to the manufacturer’s protocols. MH7A cells were seeded in 96-well plates and treated with a range of 18β-GA (50, 100, 200, 300, and 400 μM) at different time points (12, 24, 48, and 72 h). For the cell proliferation assay, CellTiter 96 Aqueous One solution was added into the 96-well plate directly and incubated at 37°C for 2 h. Absorbance was determined at 490 nm by using a termomax microplate reader.

### Cell Apoptosis Analysis

Annexin V/propidium iodide (PI) staining was used to quantify cell apoptosis. After treatments, Annexin V/PI staining was performed on MH7A cells using an Annexin V-fluorescein isothiocyanate (FITC) Apoptosis Detection kit (BioVision, Inc. Milpitas, United States) by following the manufacturer’s instructions. The fluorescence was measured by flow cytometry using Beckman flow cytometers.

### Cell Cycle Analysis

After treating with 18β-GA, cells were harvested and collected for cell cycle analysis by using the cell cycle staining kit (MutiSciences, Hangzhou, CH). In brief, cells were washed once with PBS and then stained with 1 ml buffer A and 10 μl buffer B in the cell cycle staining kit, vortexed, and kept at room temperature 30 min before flow cytometry analysis. A total of 1 × 10^4^ cells/sample were measured by using the Beckman flow cytometers. Data were analyzed using ModFit software.

### Real-Time Quantitative PCR

Real-time quantitative PCR was conducted as previously described (Huang et al., 2014). Real-time quantitative PCR was administrated using the CFX96 Touch Deep WellTM Real-Time PCR Detection System (Bio-Rad Laboratories, Inc., Berkeley, United States). The 2^−ΔΔCT^ method is employed to calculate the fold change of target gene expression. ACTB of humans and mice were used as reference, genes and the ACTB of humans was bought from Songon Biotech Co., Ltd (cat. no. B661102-0,001; Shanghai, China). Other gene sequences were obtained by PrimerBank. The primers were synthesized by Sangon Biotech Co., Ltd. (Table 1).

**TABLE 1:** Primers list for RT-qPCR.

Gene	Source	Gene source sequence (5–3′)
IL-6	Human	F: 5′-CAG​TTG​CCT​TCT​CCC​TGG​G-3′
		R: 5′-ATG​TTA​CTC​TTG​TTA​CAT​G-3′
IL-1β	Human	F: 5′-TAC​AGC​AAG​GGC​TTC​AGG -3′
		R: 5′-TCG​TAC​AGG​TGC​ATC​GTG-3′
COX-2	Human	F: 5′-CCCTTG GGTGTCAAAGGTAA-3′
		R: 5′-GCC​CTC​GCT​TAT​GAT​CTG​TC-3′
FOXO3	Human	F: 5′-CGG​ACA​AAC​GGC​TCA​CTC​T-3′
		R: 5′-CGG​ACA​AAC​GGC​TCA​CTC​T-3′
Ki67	Human	F: 5′-ACG​CCT​GGT​TAC​TAT​CAA​AAG​G-3′
		R: 5′-CAG​ACC​CAT​TTA​CTT​GTG​TTG​GA-3′
IL-6	Mouse	F: 5′-CTG​CAA​GAG​ACT​TCC​ATC​CAG-3′
		R: 5′-AGT​GGT​ATA​GAC​AGG​TCT​GTT​GG-3′
ACTB	Mouse	F: 5′-AAG​GCC​AAC​CGT​GAA​AAG​AT-3′
		R: 5′-CGC​TTC​ACG​AAT​TTG​CGT​GTC​AT-3′
IL-1β	Mouse	F: 5′-GAA​ATG​CCA​CCT​TTT​GAC​AGT​G-3′
		R: 5′-TGG​ATG​CTC​TCA​TCA​GGA​CAG-3′
COX-2	Mouse	F: 5′-TGA​GGC​AGA​AAG​AGG​TCC​AGC​CTT-3′
		R: 5′-ACC​AAT​ACT​AGC​TCA​ATA​AGT​GAC-3′
FOXO1	Mouse	F: 5′-CCC​AGG​CCG​GAG​TTT​AAC​C-3′
		R: 5′-CCC​AGG​CCG​GAG​TTT​AAC​C-3′
FOXO3	Mouse	F: 5′-CTG​GGG​GAA​CCT​GTC​CTA​TG-3′
		R: 5′-TCA​TTC​TGA​ACG​CGC​ATG​AAG-3′
FOXO4	Mouse	F: 5′-TCA​TTC​TGA​ACG​CGC​ATG​AAG-3′
		R: 5′-ACA​GGA​TCG​GTT​CGG​AGT​GT-3′

### Western Blot Analysis

The total protein was extracted from RAW264.7 cells using a RIPA Lysis Buffer (Termo Fisher Scientifc, Inc.) supplemented with PMSF and protease inhibitors. The PierceTM BCA Protein Assay Kit (Termo Fisher Scientifc, Inc.) was applied to detect protein concentrations. Western blot experiment was performed as previously described (Huang et al., 2014). Briefly, equal amounts of protein (30 μg) were separated by SDS-PAGE (10% acrylamide gel) and transferred to the PVDF membrane. Subsequently, the membranes were blocked in a solution of 5% skim milk and 0.1% Tween-20 in tris-buffered saline (TBST) for 2 h at room temperature to inhibit nonspecific protein binding. Later on, the membranes were incubated with a set of primary antibodies, including ERK1/2 antibody (cat. no. 4370P; Cell Signaling Technology, Boston, MA, United States, 1:1,000), phosphorylated ERK1/2 antibody (cat. no. 4695T; Cell Signaling Technology, 1:1,000), phosphorylated IKKα/β (*p*-IKKα/β) antibody (cat. no. 2697P; Cell Signaling Technology, 1:1,000), NF-κB2 antibody (p100/p52) (cat. no. 3017P; Cell Signaling Technology, 1:1,000), NF-κB1 antibody (p105/p50) (cat. no. 12540P; Cell Signaling Technology, 1:1,000), phosphorylated p65 antibody (p-P65) (cat. no. 3033P; Cell Signaling Technology, 1:1,000), and tubulin-α antibody (cat. no. 2125S; Cell Signaling Technology, 1:1,000). Three independent experiments were completed for each blot. Data quantification was performed using Image Lab software (Bio-Rad, Philadelphia, PA, United States).

### Statistical Analysis

The data were analyzed by using GraphPad Prism eight Software (GraphPad Software, Inc. La Jolla, United States) and presented as mean ± SD or mean ± SEM. Data were analyzed by two-way analysis of variance (ANOVA), one-way analysis of variance (ANOVA) and Student’s t-test. *p* < 0.05 was considered statistically significant.

## Results

### 18β-GA Attenuates Inflammation by Regulating the MAPK/NF-κB Signaling Pathway in MH7A and RAW264.7 Cells

**FIGURE 1:**
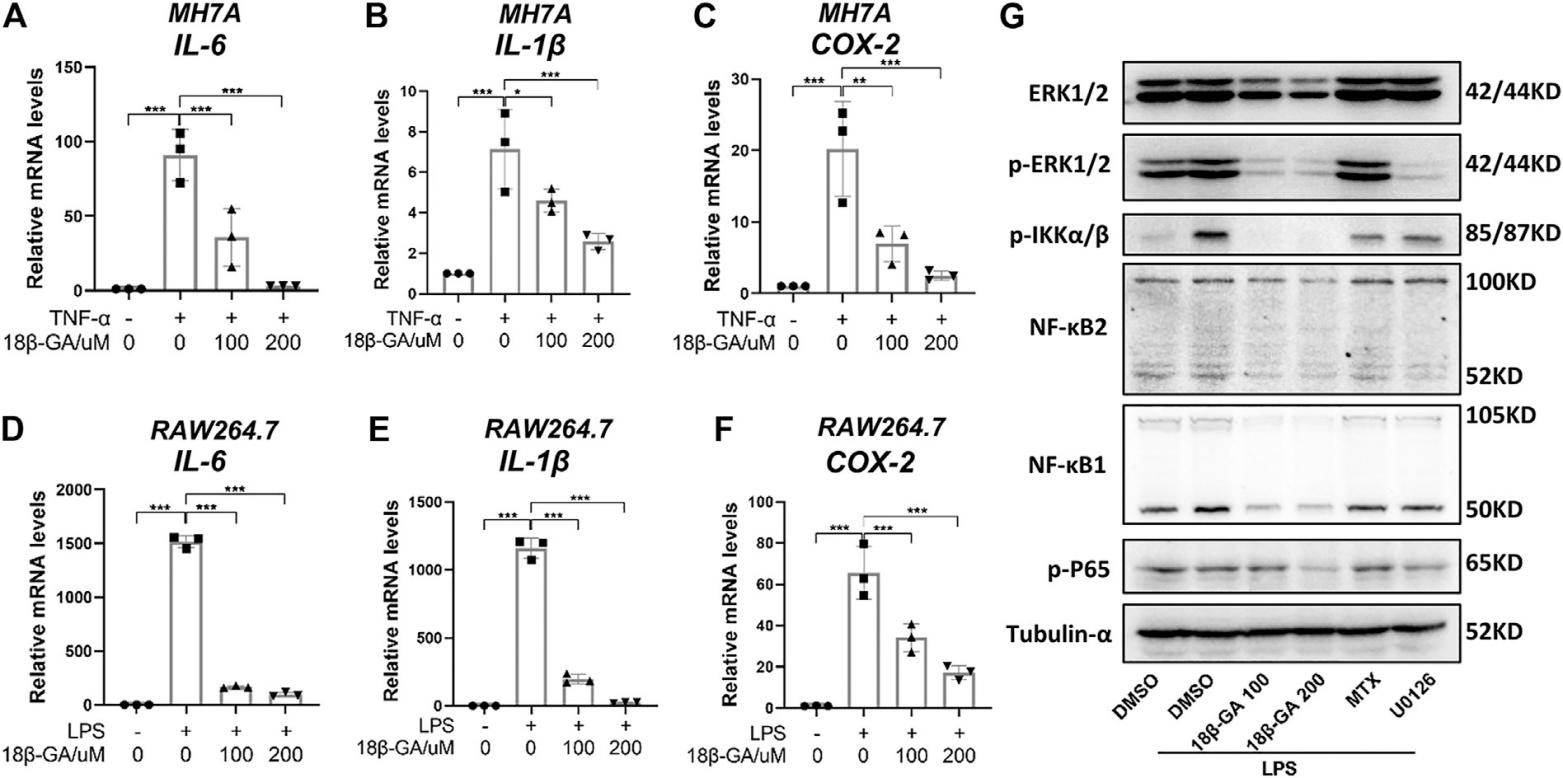
18β-GA attenuates inflammation by regulating the MAPK/NF-κB signaling pathway. **(A–C)** MH7A cells were pretreated in DMEM without FBS overnight and then the cells were exposed to TNF-α (100 ng/ml) and 18β-GA (100 and 200 μM) for 3 h. Relative mRNA levels of IL-6, IL-1β, and COX-2 were measured by RT-qPCR in MH7A cells. Data are shown as mean ± SD. ^*^, ^**^, and ^***^ mean *p* < 0.05, *p* < 0.01, and *p* < 0.001, *n* = 3. **(D–F)** RAW264.7 cells were pretreated in DMEM without FBS overnight and then the cells were exposed to LPS (1 μg/ml) and 18β-GA (100 and 200 μM) for 3 h. Relative mRNA levels of IL-6, IL-1β, and COX-2 were measured by RT-qPCR in RAW264.7 cells. Data are shown as mean ± SD. ^***^ *p* < 0.001, *n* = 3. **(G)** The protein expression of ERK1/2, *p*-ERK1/2, *p*-IKKα/β, NF-κB2, NF-κB1, and p-P65 in RAW264.7 cells were analyzed by western blot, and tubulin-α was used as house control (*n* = 3).

To evaluate the anti-inflammatory effect of 18β-GA on synoviocytes and macrophages, we established a TNF-α-stimulated MH7A cell model and an LPS-stimulated RAW264.7 cell model, respectively. Obviously, TNF-α induced MH7A cells expressing high levels of IL-6 (Figure 1A) and IL-1β (Figure 1B), both of which were inhibited by 18β-GA at the concentrations of 100 and 200 μM, which is based on Wang et al.'s study results (Wang et al., 2011; Fu et al., 2013; Chen et al., 2018). Consistent with this, another inflammatory indicator COX-2 was raised by TNF-α stimulation in MH7A cells and reduced upon 18β-GA in a dose-dependent manner (Figure 1C). Furthermore, 18β-GA showed a similar impact on IL-6, IL-1β, and COX-2 in the LPS-stimulated RAW264.7 cells (Figures 1D–F).

With the aim to explore the underlying molecular mechanism, we focus on the MAPK/NF-κB pathway, which has been illustrated to be regulated by 18β-GA in other cell lines (Cai et al., 2017; Ma et al., 2016; Mahmoud et al., 2017). In this study, methotrexate (MTX), a first-line drug for RA treatment, and U0126, an inhibitor of MEK1/2, were used as positive controls. The western blot results in Figure 1G clearly illustrated that 18β-GA treatment could inhibit the phosphorylation level of ERK1/2 stimulated by LPS in RAW264.7 cells. Later on, four key players of NF-κB signaling were measured and the result was displayed in Figure 1G, showing that the active form of NF-κB1 (P52) and NF-κB2 (P50), as well as the phosphorylation of P65 and IkappaB kinase (IKK) α/β, were all inhibited by 18β-GA. The dramatic suppression of IKK α/β may somehow explain how 18β-GA has a wide inhibition on NF-κB family members because the IKK complex is the signal integration hub for NF-κB activation (Hinz and Scheidereit, 2014). In addition, for all detected proteins, we found that 18β-GA had a more noteworthy effect than MTX and for same even better than U0126 (Figure 1G). All these results suggest that the MAPK/NF-κB pathway, at least in part, gets involved in the anti-inflammatory mechanism of 18β-GA against RA.

### 18β-GA Treatment Decreases Cell Viability and Cell Proliferative Capacity

**FIGURE 2:**
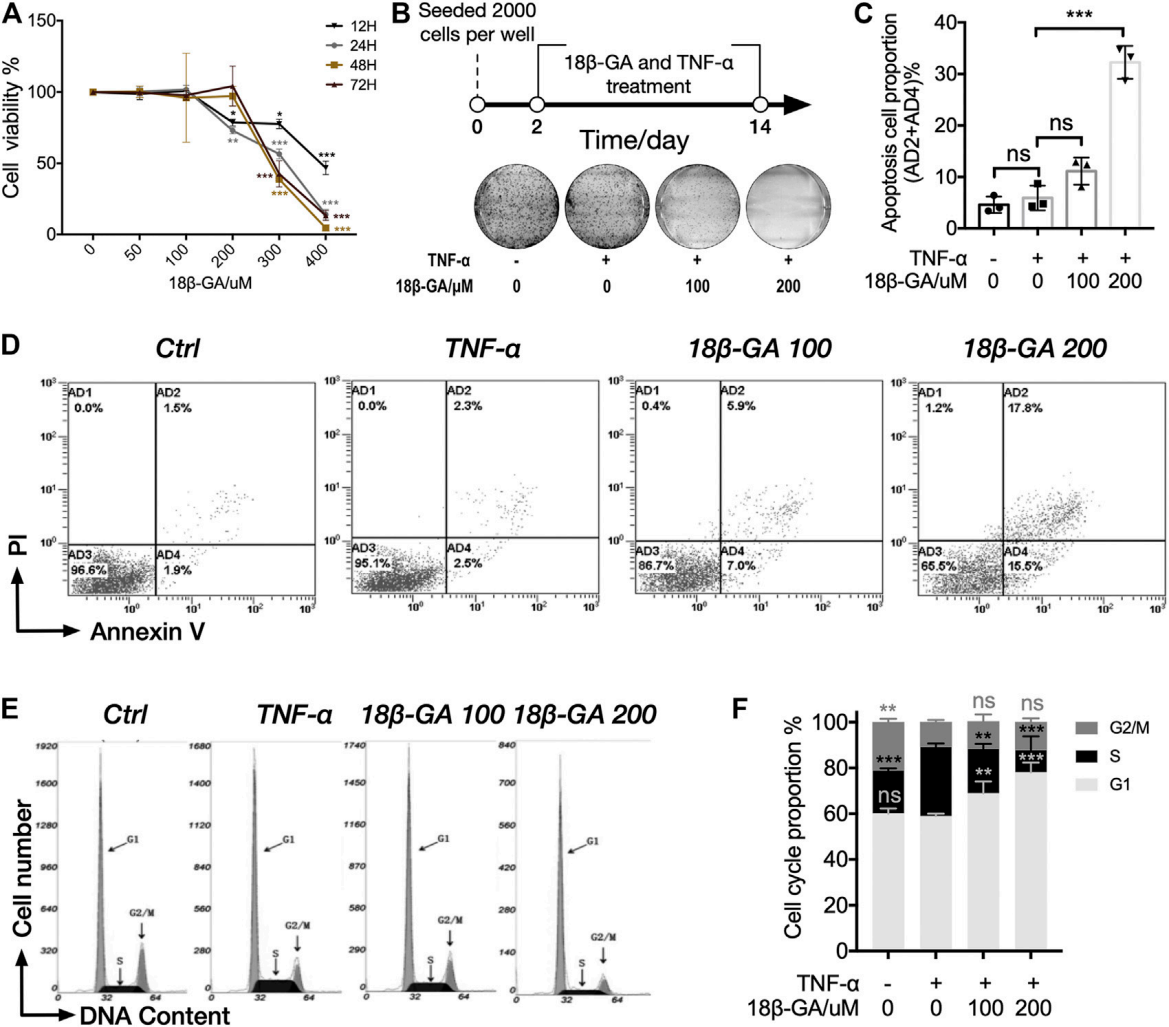
18β-GA suppresses cell proliferation through inducing cell apoptosis and cell cycle arrest. **(A)** MH7A cells were treated with gradients of 18β-GA along with 100 ng/ml TNF-α for different time periods (12–48 h), and cell viability was detected by the MTS assay. Significance was determined by using two-way ANOVA with Tukey testing, and error bars indicate SD. ^*^ *p* < 0.05; ^**^ *p* < 0.01; ^***^ *p* < 0.001, *n* = 3. **(B)** The colony formation assay was performed for the determination of cytotoxicity of 18β-GA. A simplified workflow and representative result are shown (*n* = 3). **(C)** Quantification of the data in D. Sum of AD2 and AD4 is calculated and presented in the graph. One-way ANOVA with Tukey test is applied for statistical analysis, error bars indicate SD, and ns means no significant change; ^***^ *p* < 0.001, *n* = 3. **(D)** The cell apoptosis assay is stained with PI and Annexin V and measured by flow cytometry. Representative flow cytometry plots are shown (*n* = 3). (E) Representative histogram of MH7A cells stained with nuclear dye showing DNA content distribution. G1, S, and G2/M peaks are highlighted by arrow. **(F)** Quantification result of the cell cycle assay. Data show mean ± SD, as comparing with TNF-α stimulation, and ns means no significant change; ^**^ *p* < 0.01; ^***^ *p* < 0.001, n = 3.

The MTS assay was adopted to measure the influence of 18β-GA on cell viability in MH7A cells. MH7A cells were treated with different doses of 18β-GA from 0 to 400 µM with 100 ng/ml TNF-α. Data shown in Figure 2A demonstrated that 18β-GA treatment reduced MH7A cell viability in a dose-dependent manner (Figure 2A). Specifically, 18β-GA showed significant inhibition on cell viability from a dose of 200 µM in 12 and 24 h time courses. The half-maximal inhibitory concentrations (IC50) of 18β-GA are 392.96, 310.69, 280.475, and 242.99 µM in 12, 24, 48, and 72 h, respectively. According to the IC50 and previous studies, the concentration of 18β-GA (100 µM or 200 µM) was performed in MH7A cells for next experiments (Figure 2A) (Wang et al., 2011; Fu et al., 2013; Chen et al., 2018). Moreover, we observed that 18β-GA (100 and 200 µM) suppressed cell colony formation in a colony-forming assay using crystal violet stain (Figure 2B), indicating that 18β-GA has a negative impact on cell proliferative capacity.

### 18β-GA Treatment Induces Cell Apoptosis and Cell Cycle Arrest

Furthermore, we carried out the cell apoptosis assay and cell cycle assay by using cytometry in order to understand how 18β-GA suppresses cell proliferative capacity. In both assays, MH7A cells were treated with 0 μM, 100 µM, or 200 µM of 18β-GA accompanied with 100 ng/ml TNF-α for 24 h. Cells without administration of TNF-α and 18β-GA were used as control. As illustrated in Figures 2C,D, the proportion of apoptotic cells (AD2+AD4%) was significantly higher in cells treated with 18β-GA (about 2.5 and 7 times for 100 and 200 μM of 18β-GA treatment, respectively, as compared to TNF-α treatment). Data showed in Figure 2F summarized the cell cycle assay results from three independent experiments. The percentage of cells in the G1 phase increased from 58.38% (cells only stimulated with TNF-α) up to 68.95 and 77.81% after 24 h treatment of 100 and 200 μM 18β-GA. In addition, TNF-α stimulation significantly increased the proportion of S phase cells from 19.00 to 30.86%, which can be restored by treating cells with 18β-GA (proportion of S phase cells are 19.44 and 9.88% in 100 and 200 μM 18β-GA treatment, respectively).

### The Dysregulation of FOXO Family in the Synovial Tissue of RA Patients Is Identified in the Study of Online Microarray Datasets

**FIGURE 3:**
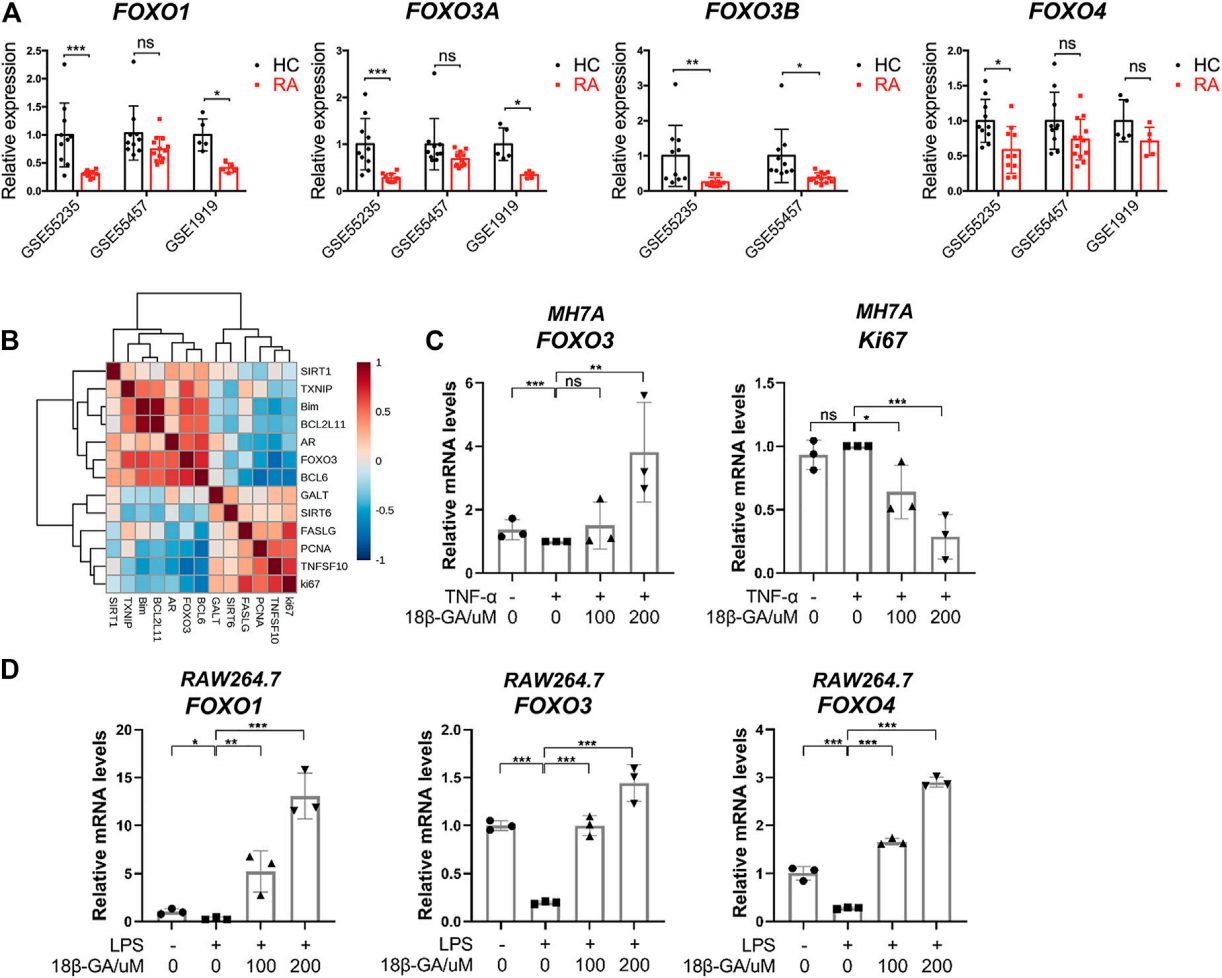
The dysregulation of the FOXO family in the synovial tissue of RA and the effects of 18β-GA on the FOXO pathway. **(A)** The relative expression of FOXO1, FOXO3a, FOXO3b, and FOXO4 in the synovial tissue of healthy people (HC) and rheumatoid arthritis patients (RA). Data are extracted from online microarray datasets, including GSE55235 (*n* = 10 for HC and RA), GSE55457 (*n* = 10 for HC and RA), and GSE 1919 (*n* = 5 for HC and RA). Data are shown as mean ± SD. ns means no significant change; ^*^ *p* < 0.05; ^***^ *p* < 0.001. **(B)** Correlation heatmap of indicated genes in the synovial tissue of joint from RA patients (GSE55235). **(C)** Relative mRNA levels of FOXO3 and Ki67 were detected by RT-qPCR in MH7A cells. Data are shown as mean ± SD. ns, ^*^, ^**^, and ^***^ mean no significant change, *p* < 0.05, *p* < 0.01, and *p* < 0.001, *n* = 3. **(D)** Relative mRNA levels of FOXO1, FOXO3, and FOXO4 were detected by RT-qPCR in RAW264.7 cells. Data are shown as mean ± SD. ns, ^*^, ^**^, and ^***^ mean no significant change, *p* < 0.05, *p* < 0.01, and *p* < 0.001, *n* = 3.

To reveal the role of FOXO3 in the pathogenesis of RA, the expression levels of FOXO family members were examined in the RA synovial tissue based on data from three online genome-wide transcriptomic datasets (the accession numbers are GSE55235, GSE55457, and GSE1919, respectively). Since multiple probes were used for the detection of FOXO1, FOXO3a, and FOXO3b in the microarray platform, we averaged the value detected by different probes for each gene and then calculated the relative expression level in healthy control (HC) and RA. It is notable that all members of the FOXO family have a lower expression in the RA synovial tissue than the healthy synovial tissue. In GSE55235 and GSE 1919, the expression patterns of FOXO1 and FOXO3a in the RA synovial tissue are similar, but there is no significant decrease in the RA synovial tissue of GSE55457. Though there is no probe for the detection of FOXO3b in GSE 1919, other datasets demonstrated the same result that FOXO3b was decreased in the RA synovial tissue significantly. However, the difference between HC and RA on FOXO4 is not obvious (Figure 3A). The results suggest that the reduction of FOXO3 may play a pathological role in RA. To confirm this conclusion, we moved our attention to those well-studied target genes of FOXO3. The correlation analysis of FOXO3a and a set of genes were shown in the heatmap, demonstrating that FOXO3a was positively correlated to its target genes. In particular, genes that participate in the apoptotic process, such as Bim, BCL2L11, and BCL6, were also negatively correlated with the proliferation marker gene (Ki67) (Figure 3B). Together, these data indicate the contribution of FOXO-dependent signaling to synoviocyte hyperproliferation, thereby highlighting the potential of therapeutically exploiting FOXO3-dependent RA pathogenesis.

### The FOXO3 Is Increased by Administration of 18β-GA in MH7A and RAW264.7 Cells

Since FOXO3 plays a pivotal role in the regulation of cell proliferation and apoptosis, we then tested if the cell apoptosis and cell cycle arrest induced by 18β-GA were dependent on the modulation of FOXO3 signaling. As illustrated in Figure 3C, the mRNA level of FOXO3 was mildly declined after TNF-α addition but then significantly raised by 200 μM of 18β-GA treatment in MH7A cells. On the contrary, ki67, the cell proliferation marker, was decreased by 18β-GA in a dose-dependent manner (Figure 3C). Unsurprisingly, we observed the same effect of 18β-GA on FOXO signaling in RAW264.7 cells. As shown in Figure 3D, the LPS-induced suppression of mRNA levels of FOXO1, FOXO3, and FOXO4 was dramatically increased by 100 and 200 μM administrations of 18β-GA (Figure 3D).

### 18β-GA Administration Attenuates Arthritis in Collagen-Induced Arthritis Animal Models

**FIGURE 4:**
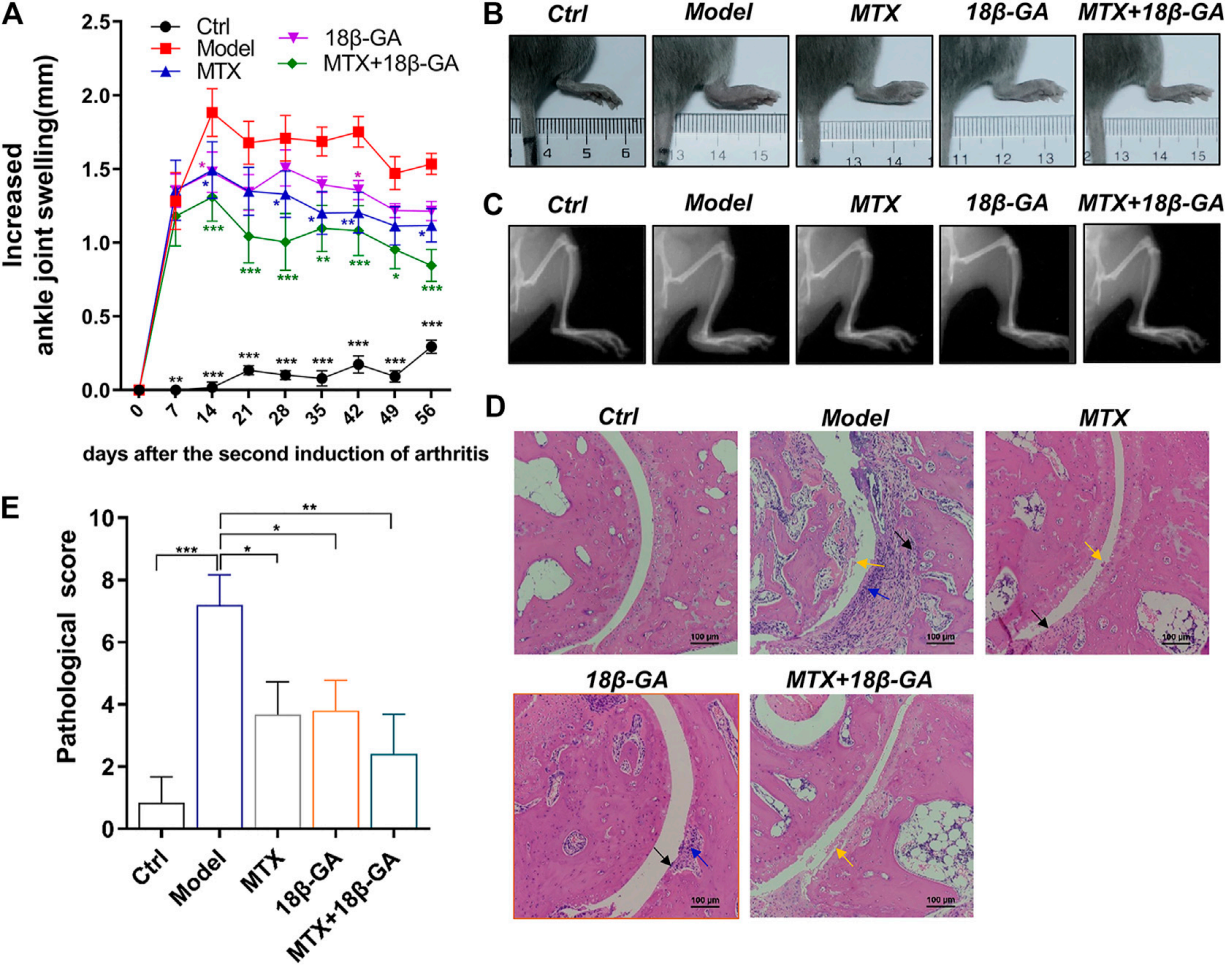
18β-GA administration attenuates arthritis severity in the CIA model. **(A)** The increased ankle joint volume is shown in A. Data are shown as mean ± SEM. Two-way ANOVAD was used to analyze differences between groups, ^*^ *p* < 0.05, ^**^ *p* < 0.01, ^***^ *p* < 0.001 (compared with the model group), *n* = 6. **(B, C)** The photographs and X-ray images of ankles are shown in B and C. **(D)** Representative images of pathological and sections of ankle in mice in different groups are shown in D. Red arrow indicates inflammatory cell infiltration, black arrow indicates cartilage hyperplasia, and yellow arrow indicates bone and cartilage destruction and erosion. **(E)**The pathological improvements are assessed by pathological score. Data are shown as mean ± SEM.

To explore the therapeutic effect of 18β-GA for RA, a commonly used CIA model was adopted and orally administered with 18β-GA. MTX treatment was used as a positive control. The body weights of mice in different groups were evaluated, and the results showed that there was no significant difference after collagen induction and 18β-GA and MTX administration (Supplemental Figure S1A). The level of ankle joint swelling was used as a direct indicator to evaluate RA. Notably, the CIA model was established successfully, as evidenced in Figure 4A, and the ankle joint of mice in the model group is much swelling than the control. However, after 18β-GA and MTX intervention, the mice showed less aggravated symptoms, as assessed by ankle joint volumes. In particular, the decrease in swelling was obviously observed in the combined group of 18β-GA and MTX. Representative images of joints are exhibited in Figure 4B, showing that 18β-GA, MTX, and 18β-GA + MTX could reduce joint swelling of CIA mice. In addition, the X-ray was used to evaluate the effect of 18β-GA on bone erosion. The data shown in Figure 4C demonstrated that bone erosion was diminished in the ankle of the 18β-GA-treated mice as compared to the vehicle-treated animals. To confirm the therapeutic effect of 18β-GA, H&E staining of the ankle joint was performed. Compared with control mice, the ankle section of CIA mice exhibited severe inflammatory cell infiltration (blue arrow), cartilage hyperplasia (black arrow), bone and cartilage destruction, and erosion (yellow arrow, Figure 4D). The pathological score was then assessed and displayed in Figure 4E and was improved in 18β-GA, MTX, and 18β-GA + MTX treated animals compared to the vehicle-treated animals, especially in the combined group of 18β-GA and MTX. Moreover, it was apparent that the pathological state of the ankle joint tissues was partly improved in the 18β-GA, MTX, and 18β-GA + MTX groups, which further confirmed the therapeutic effect of 18β-GA. These results indicate that 18β-GA has the same curative effect as MTX for the treatment of RA, and the combination of both can have a better effect.

### 18β-GA Treatment Decreases the Serum Levels of Proinflammatory Cytokines and Attenuates MTX-Derive Liver Damage

**FIGURE 5:**
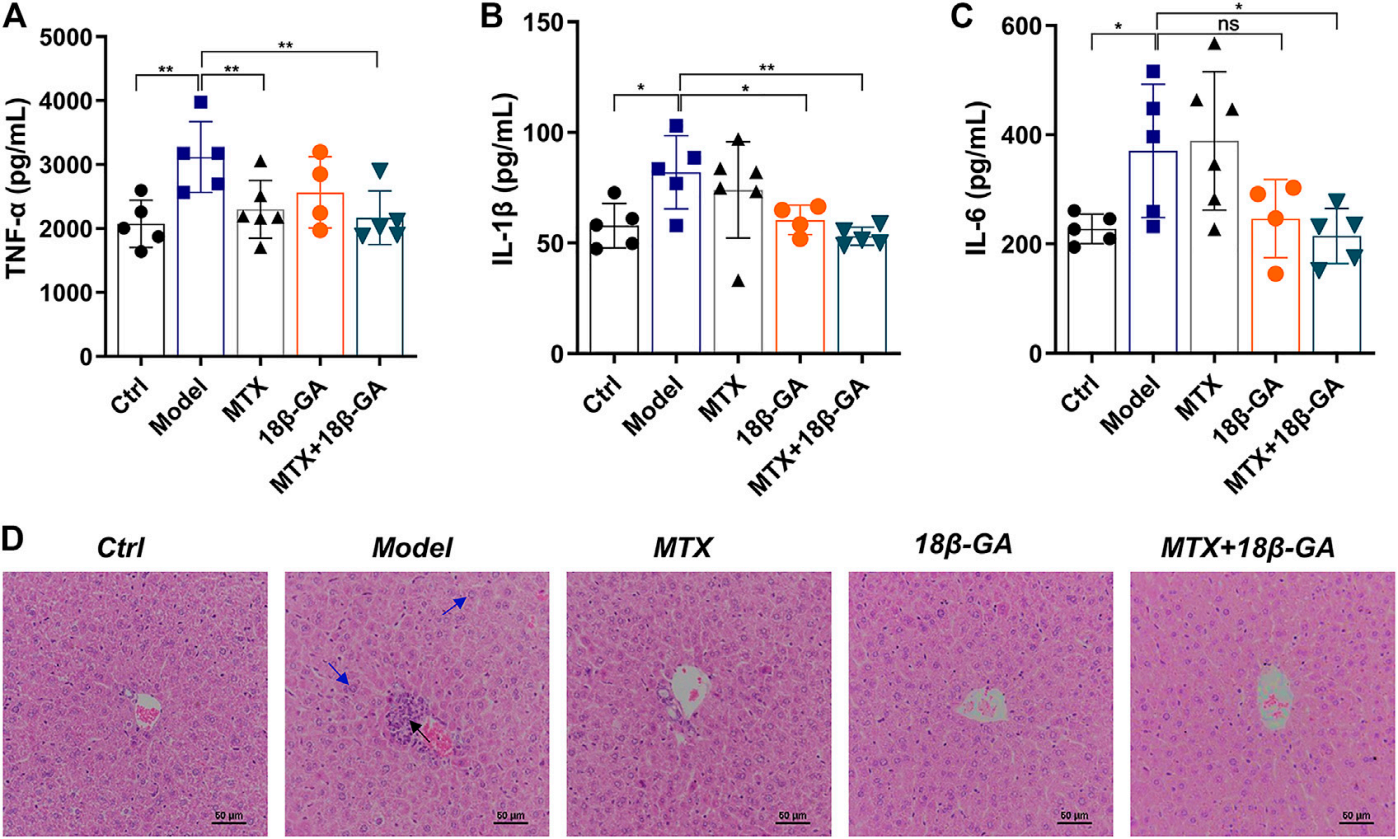
18β-GA attenuates secretion of serum cytokines and liver pathological injury. **(A–C)** Serum levels of TNF-α, IL-1β, and IL-6 were measured by ELISA. Data are shown as mean ± SD. ^*^ *p* < 0.05; ^**^ *p* < 0.01; ^***^ *p* < 0.001, *n* = 4–6. **(D)** Representative images of pathology and sections of the liver in mice in different groups are shown in D. Black arrow indicates portal inflammation, and blue arrow indicates hepatocyte swelling and loosening.

As demonstrated above, TNF-α, IL-1β, and IL-6 are potential therapeutic targets of 18β-GA. With the aim to further confirm these conclusions, we detected all these three cytokines in the serum of mice in different groups by ELISA. As shown in Figures 5A–C, the serum levels of TNF-α, IL-1β, and IL-6 were up-regulated in CIA mice as compared to the control group, while these elevations were decreased after the administration of either 18β-GA or 18β-GA combined with MTX. (Figures 5A–C). Since a part of RA patients treated with MTX often suffered various degrees of liver damage, it is worth investigating if 18β-GA has hepatoprotective activity. To reach this aim, we applied H&E staining of liver sections of mice in all six groups, and the representative pictures were exhibited in Figure 5D. The liver section in the control group contained a normal lobular structure and radiating hepatic cords, without necrosis or inflammatory infiltration. By contrast, liver sections of mice in the CIA group and MTX group displayed typical pathological characteristics of liver injury, like portal inflammation (highlighted by black arrow in Figure 5D) and hepatocyte swelling and loosening (highlighted by blue arrow in Figure 5D). Notably, 18β-GA administration showed potential protective effects in either collagen- or MTX-derived liver damage by reducing inflammatory cell infiltration and hepatocyte swelling (Figure 5D). These results suggest that 18β-GA treatment has the benefits of not only attenuating the expression of pro-inflammatory cytokines but also protecting against liver injury.

## Discussion

RA is recognized as a chronic autoimmune disease characterized by inflammation of joints and hyperplasia of fibroblast-like synoviocytes (FLSs) (Bottini and Firestein, 2013). Currently, the treatment strategies for RA mainly focus on specifically targeting inflammatory mediators secreted by FLS and immune cells or protecting the synovium from inflammation (Noss and Brenner, 2008). 18β-GA, an active ingredient of Licorice, may be a valuable candidate for RA therapy. Previous studies reported that both glycyrrhetinic acid (GA) and 18β-GA manifest anti-inflammatory activities through different mechanisms, for example, inhibited MAP kinase or PI3K/Akt/GSK3β signaling pathways (Kao et al., 2010a; Ukil et al., 2011). Specifically, the activities and expressions of three phosphatases MKP1, MKP3, and protein phosphatase 2A (PP2A) were proved to be significantly inhibited by 18β-GA, and the dephosphorylation of p38 and ERK in infected bone marrow–derived macrophages (BMDM) were also significantly inhibited by 18β-GA (Ukil et al., 2011). However, the anti-inflammatory effects and mechanisms of 18β-GA against fibroblast-like synoviocytes remain unexplained.

In this study, two cell models were established. They are TNF-α induced MH7A cells and LPS stimulated RAW264.7 cells. We found that 18β-GA reduced the expression of inflammatory cytokines in RAW264.7 cells, which is consistent with the results of other studies (Kao et al., 2010a; Ukil et al., 2011; Yang et al., 2017). Interestingly, 18β-GA also exhibited a significant inhibitory effect on TNF-α-induced production of IL-1β, IL-6, and COX-2 in MH7A cells. Moreover, we found that the effects of 18β-GA to alleviate inflammation of RAW264.7 cells were achieved through regulating the MAPK/NF-κB signaling pathway. Thus, 18β-GA can identify a critical factor that targets inflammation mediated by MH7A and RAW264.7 cells, which may provide a wide array of potential drug candidates to help long-term remission for RA.

In addition, previous studies implied that 18β-GA had negative impacts on cell proliferation and growth. For example, 18β-GA inhibits cell proliferation by suppressing thromboxane synthase in non-small-cell lung cancer (Kao et al., 2010a; Ukil et al., 2011; Yang et al., 2017), induces cell apoptosis via induction of the ROS/MAPKs-mediated pathway in pituitary adenoma (Wang et al., 2014), and potentiates the Hsp90 inhibition-induced apoptosis in ovarian carcinoma (Yang et al., 2012). Therefore, we attempted to explore the role of 18β-GA on cell proliferation in RA. First, we found 18β-GA reduced cell viability in a dose-dependent manner and inhibited colony-forming capacity in MH7A cells (Figures 2A,B). Then, we demonstrated that 18β-GA could cause G1 phase cell cycle arrest and induce cell apoptosis in MH7A cells (Figures 2C–F). These observations suggest an effect of 18β-GA in suppressing cell proliferation of MH7A cells, which may link to a potential mechanism of 18β-GA that suppresses FOXO3 and leads to the initiation of the cell apoptosis process (Obexer et al., 2007; Weng et al., 2008).

Interestingly, after digging into the data of three online microarray studies, we have for the first time uncovered the down-expression of members of the FOXO3 family in the RA synovial tissue (Figure 3A). FOXO3, a protein that belongs to the O subclass of the forkhead family of transcription factors, is a well-known tumor suppressor. It may trigger apoptosis by upregulating genes required for cell death, such as BCL2L11 and Bim (You et al., 2006), or downregulating anti-apoptotic proteins, such as FLIP (Skurk et al., 2004). We observed that 18β-GA up-regulated FOXO3 (Figure 3B) and down-regulated the cell proliferation marker ki67 in MH7A cells (Figure 3C). Since inflammatory processes are very prominent in a number of macrophage-related diseases, targeting macrophages may be an ideal strategy for treating inflammation and diseases. For example, for RA, LPS stimulation can directly promote the differentiation of macrophage RAW264.7 osteoclasts and inhibit osteoclast apoptosis, thereby aggravating inflammation and bone destruction (Huang et al., 2019). Thus, FOXO1, FOXO2, and FOXO4 have also been detected in macrophage RAW264.7 cells, and the result showed that 18β-GA treatment increased the mRNA levels of FOXO1, FOXO2, and FOXO4 (Figure 3D). All evidence suggesting 18β-GA restored the imbalance between proliferation and apoptosis through mediating the FOXO pathway in RA.

Meanwhile, accumulating evidence indicates that 18β-GA has a protective effect on both inflammation and chronic inflammatory conditions including RA (Kim et al., 2010). In this study, we found that 18β-GA, especially 18β-GA combined with MTX, dramatically reduced collagen-induced arthritis as well as TNF-α- and LPS-induced cell inflammation (Figure 4B; Figures 1A,B). In addition, 18β-GA and 18β-GA combined with MTX also obviously reduced inflammatory cell infiltration in the liver (Figure 5B). The mechanism study showed that 18β-GA inhibited the activity of the MAPK/NF-κB pathway (Figure 1C). In line with our study, it has been shown that 18β-GA modulated LPS-induced inflammation by inhibiting NF-κB through decreasing PI3K p110delta and p110gamma (Wang et al., 2011). A recent study further revealed that 18β-GA treatment led to the dissociation of a glucocorticoid receptor (GR)-HSP90 complex, thereby blocking inflammation (Kao et al., 2010a).

In summary, we highlighted the pivotal role of MAPK/NF-κB and FOXO3 in 18β-GA mediated anti-inflammation and anti-proliferation effects, respectively. Now, there is a global attempt to look for a potential drug for the treatment of RA with low toxicity and high efficiency. The research we presented here suggests that 18β-GA could be an ideal candidate for RA therapy.

## Data Availability

The original contributions presented in the study are included in the article/Supplementary Material; further inquiries can be directed to the corresponding authors.

## References

[B1] Bottini N. Firestein G. S. (2013). Duality of Fibroblast-like Synoviocytes in RA: Passive Responders and Imprinted Aggressors. Nat. Rev. Rheumatol. 9 (1), 24–33. 10.1038/nrrheum.2012.190 23147896 PMC3970924

[B2] Brzustewicz E. Bryl E. (2015). The Role of Cytokines in the Pathogenesis of Rheumatoid Arthritis - Practical and Potential Application of Cytokines as Biomarkers and Targets of Personalized Therapy. Cytokine 76 (2), 527–536. 10.1016/j.cyto.2015.08.260 26321413

[B3] Cai Y. Zhao B. Liang Q. Zhang Y. Cai J. Li G. (2017). The Selective Effect of Glycyrrhizin and Glycyrrhetinic Acid on Topoisomerase IIα and Apoptosis in Combination with Etoposide on Triple Negative Breast Cancer MDA-MB-231 Cells. Eur. J. Pharmacol. 809, 87–97. 10.1016/j.ejphar.2017.05.026 28506909

[B4] Chen J. Zhang Z.-q. Song J. Liu Q.-m. Wang C. Huang Z. (2018). 18β-Glycyrrhetinic-acid-mediated Unfolded Protein Response Induces Autophagy and Apoptosis in Hepatocellular Carcinoma. Sci. Rep. 8 (1). 10.1038/s41598-018-27142-5 PMC6008326 29921924

[B5] Conigliaro P. Triggianese P. De Martino E. Fonti G. L. Chimenti M. S. Sunzini F. (2019). Challenges in the Treatment of Rheumatoid Arthritis. Autoimmun. Rev. 18 (7), 706–713. 10.1016/j.autrev.2019.05.007 31059844

[B6] Firestein G. S. (2005). Immunologic Mechanisms in the Pathogenesis of Rheumatoid Arthritis. JCR: J. Clin. Rheumatol. 11, S39–S44. 10.1097/01.rhu.0000166673.34461.33 16357749

[B7] Frisell T. Saevarsdottir S. Askling J. (2016). Family History of Rheumatoid Arthritis: an Old Concept with New Developments. Nat. Rev. Rheumatol. 12 (6), 335–343. 10.1038/nrrheum.2016.52 27098907

[B8] Fu X.-X. Du L.-L. Zhao N. Dong Q. Liao Y.-H. Du Y.-M. (2013). 18β-Glycyrrhetinic Acid Potently Inhibits Kv1.3 Potassium Channels and T Cell Activation in Human Jurkat T Cells. J. Ethnopharmacology 148 (2), 647–654. 10.1016/j.jep.2013.05.022 23707333

[B9] Hinz M. Scheidereit C. (2014). The IκB Kinase Complex in NF ‐κB Regulation and beyond. EMBO Rep. 15 (1), 46–61. 10.1002/embr.201337983 24375677 PMC4303448

[B10] Huang L. Chen Q. Yu L. Bai D. (2019). Pyropheophorbide-α Methyl Ester-Mediated Photodynamic Therapy Induces Apoptosis and Inhibits LPS-Induced Inflammation in RAW264.7 Macrophages. Photodiagnosis Photodynamic Ther. 25, 148–156. 10.1016/j.pdpdt.2018.12.002 30562579

[B11] Huang R.-Y. Li S.-S. Guo H.-Z. Huang Y. Zhang X. Li M.-Y. (2014). Thromboxane A2 Exerts Promoting Effects on Cell Proliferation through Mediating Cyclooxygenase-2 Signal in Lung Adenocarcinoma Cells. J. Cancer Res. Clin. Oncol. 140 (3), 375–386. 10.1007/s00432-013-1573-3 24384873 PMC11823903

[B12] Hung C.-F. Hsiao C.-Y. Hsieh W.-H. Li H.-J. Tsai Y.-J. Lin C.-N. (2017). 18ß-glycyrrhetinic Acid Derivative Promotes Proliferation, Migration and Aquaporin-3 Expression in Human Dermal Fibroblasts. PLoS One 12 (8), e0182981. 10.1371/journal.pone.0182981 28813533 PMC5558956

[B13] Kao T.-C. Shyu M.-H. Yen G.-C. (2010a). Glycyrrhizic Acid and 18β-Glycyrrhetinic Acid Inhibit Inflammation via PI3K/Akt/GSK3β Signaling and Glucocorticoid Receptor Activation. J. Agric. Food Chem. 58 (15), 8623–8629. 10.1021/jf101841r 20681651

[B14] Kim K. R. Jeong C.-K. Park K.-K. Choi J.-H. Park J. H. Y. Lim S. S. (2010). Anti-Inflammatory Effects of Licorice and Roasted Licorice Extracts on TPA-Induced Acute Inflammation and Collagen-Induced Arthritis in Mice. J. Biomed. Biotechnol. 2010, 1–8. 10.1155/2010/709378 PMC2841253 20300198

[B15] Klinkhoff A. (2004). Biological Agents for Rheumatoid Arthritis: Targeting Both Physical Function and Structural Damage. Drugs 64, 1267–1283. 10.2165/00003495-200464120-00001 15200343

[B16] Kosmaczewska A. Świerkot J. Ciszak L. Wiland P. (2011). Rola Subpopulacji Limfocytów Pomocniczych Th1, Th17 I Treg W Patogenezie Reumatoidalnego Zapalenia Stawów Z Uwzględnieniem Przeciwzapalnego Działania Cytokin Th1. Postepy Hig Med. Dosw 65, 397–403. 10.5604/17322693.948971 21734324

[B17] Ma T. Huang C. Meng X. Li X. Zhang Y. Ji S. (2016). A Potential Adjuvant Chemotherapeutics, 18β-Glycyrrhetinic Acid, Inhibits Renal Tubular Epithelial Cells Apoptosis via Enhancing BMP-7 Epigenetically through Targeting HDAC2. Sci. Rep. 6 (1). 10.1038/srep25396 PMC4857087 27145860

[B18] Mahmoud A. M. Hussein O. E. Hozayen W. G. Abd El-Twab S. M. (2017). Methotrexate Hepatotoxicity Is Associated with Oxidative Stress, and Down-Regulation of PPARγ and Nrf2: Protective Effect of 18β-Glycyrrhetinic Acid. Chemico-Biological Interactions 270, 59–72. 10.1016/j.cbi.2017.04.009 28414158

[B19] Majithia V. Geraci S. A. (2007). Rheumatoid Arthritis: Diagnosis and Management. Am. J. Med. 120 (11), 936–939. 10.1016/j.amjmed.2007.04.005 17976416

[B20] Mcinnes I. B. Schett G. (2011). The Pathogenesis of Rheumatoid Arthritis. N. Engl. J. Med. 365 (23), 2205–2219. 10.1056/NEJMra1004965 22150039

[B21] Noss E. H. Brenner M. B. (2008). The Role and Therapeutic Implications of Fibroblast-like Synoviocytes in Inflammation and Cartilage Erosion in Rheumatoid Arthritis. Immunol. Rev. 223, 252–270. 10.1111/j.1600-065X.2008.00648.x 18613841

[B22] Obexer P. Geiger K. Ambros P. F. Meister B. Ausserlechner M. J. (2007). FKHRL1-mediated Expression of Noxa and Bim Induces Apoptosis via the Mitochondria in Neuroblastoma Cells. Cell Death Differ 14 (3), 534–547. 10.1038/sj.cdd.4402017 16888645

[B23] Skurk C. Maatz H. Kim H.-S. Yang J. Abid M. R. Aird W. C. (2004). The Akt-Regulated Forkhead Transcription Factor FOXO3a Controls Endothelial Cell Viability through Modulation of the Caspase-8 Inhibitor FLIP. J. Biol. Chem. 279 (2), 1513–1525. 10.1074/jbc.M304736200 14551207

[B24] Smolen J. S. Aletaha D. (2015). Rheumatoid Arthritis Therapy Reappraisal: Strategies, Opportunities and Challenges. Nat. Rev. Rheumatol. 11 (5), 276–289. 10.1038/nrrheum.2015.8 25687177

[B25] Ukil A. Kar S. Srivastav S. Ghosh K. Das P. K. (2011). Curative Effect of 18β-Glycyrrhetinic Acid in Experimental Visceral Leishmaniasis Depends on Phosphatase-dependent Modulation of Cellular MAP Kinases. PLoS One 6 (12), e29062. 10.1371/journal.pone.0029062 22194991 PMC3237588

[B26] Wang C.-Y. Kao T.-C. Lo W.-H. Yen G.-C. (2011). Glycyrrhizic Acid and 18β-Glycyrrhetinic Acid Modulate Lipopolysaccharide-Induced Inflammatory Response by Suppression of NF-Κb through PI3K P110δ and P110γ Inhibitions. J. Agric. Food Chem. 59 (14), 7726–7733. 10.1021/jf2013265 21644799

[B27] Wang D. Wong H.-K. Feng Y.-B. Zhang Z.-J. (2014). 18beta-Glycyrrhetinic Acid Induces Apoptosis in Pituitary Adenoma Cells via ROS/MAPKs-mediated Pathway. J. Neurooncol. 116 (2), 221–230. 10.1007/s11060-013-1292-2 24162829

[B28] Weng S.-C. Kashida Y. Kulp S. K. Wang D. Brueggemeier R. W. Shapiro C. L. (2008). Sensitizing Estrogen Receptor-Negative Breast Cancer Cells to Tamoxifen with OSU-03012, a Novel Celecoxib-Derived Phosphoinositide-dependent Protein kinase-1/Akt Signaling Inhibitor. Mol. Cancer Ther. 7 (4), 800–808. 10.1158/1535-7163.MCT-07-0434 18413793

[B29] Yang G. Wang L. Yu X. Huang Y. Qu C. Zhang Z. (2017). Protective Effect of 18β-Glycyrrhetinic Acid against Triptolide-Induced Hepatotoxicity in Rats. Evidence-Based Complement. Altern. Med. 2017, 1–12. 10.1155/2017/3470320 PMC5440796 28572827

[B30] Yang J. C. Myung S. C. Kim W. Lee C. S. (2012). 18β-Glycyrrhetinic Acid Potentiates Hsp90 Inhibition-Induced Apoptosis in Human Epithelial Ovarian Carcinoma Cells via Activation of Death Receptor and Mitochondrial Pathway. Mol. Cel. Biochem. 370 (1-2), 209–219. 10.1007/s11010-012-1412-x 22865487

[B31] You H. Pellegrini M. Tsuchihara K. Yamamoto K. Hacker G. Erlacher M. (2006). FOXO3a-dependent Regulation of Puma in Response to Cytokine/growth Factor Withdrawal. J. Exp. Med. 203 (7), 1657–1663. 10.1084/jem.20060353 16801400 PMC2118330

