# Supplementary material for: Anti-inflammatory and Pro-apoptotic Effects of 18beta-Glycyrrhetinic Acid In Vitro and In Vivo Models of Rheumatoid Arthritis
Source: Front Pharmacol. 2021 Jul 26;12:681525. doi: 10.3389/fphar.2021.681525 (PMC8351798; doi:10.3389/fphar.2021.681525)
Supplement: Supplementary file 1 [file DataSheet2.PDF]

Manuscript ID: 681525

Title: Anti-inflammatory and pro-apoptotic effects of 18beta-glycyrrhetinic acid *in vitro* and *in vivo* models of rheumatoid arthritis.

Short Title: Effects of 18beta-glycyrrhetinic acid in rheumatoid arthritis.

Yunhui Feng1[†], Mphil; Liyan Mei[2†], Mphil; Maojie Wang[2,3], MD, Mphil; Qingchun Huang[2*], MD, PhD; Runyue Huang[2,4,5,6*], MD, PhD.

[†] These authors equally contributed to the article

Affiliations:

[1] College of physical education, Guangzhou University, Guangzhou, China

[2] The Second Affiliated Hospital of Guangzhou University of Chinese Medicine (Guangdong Provincial Hospital of Chinese Medicine), Guangzhou 510006, Guangzhou, China

[3] Center for Molecular Medicine, University Medical Center Utrecht, the Netherlands

[4] Guangdong Provincial Key Laboratory of Clinical Research on Traditional Chinese Medicine Syndrome, Guangzhou, China

[5] State Key Laboratory of Dampness Syndrome of Chinese Medicine (The Second Affiliated Hospital of Guangzhou University of Chinese Medicine), Guangzhou, China

[6] Guangdong-Hong Kong-Macau Joint lab on Chinese Medicine and Immune Disease Research, Guangzhou University of Chinese Medicine

* **Corresponding author**: Prof. Run-Yue Huang, Section Rheumatology Research, The Second Affiliated Hospital, Guangzhou University of Chinese Medicine (Guangdong Provincial Hospital of Chinese Medicine), Guangzhou, ChinaEmail: ryhuang@gzucm.edu.cn

* Co-corresponding author: Prof. Qingchun Huang, Rheumatology Department, The Second Affiliated Hospital, Guangzhou University of ChineseMedicine (Guangdong Provincial Hospital of ChineseMedicine), Guangzhou, ChinaEmail: qch1963@163.com

First raw WB data:

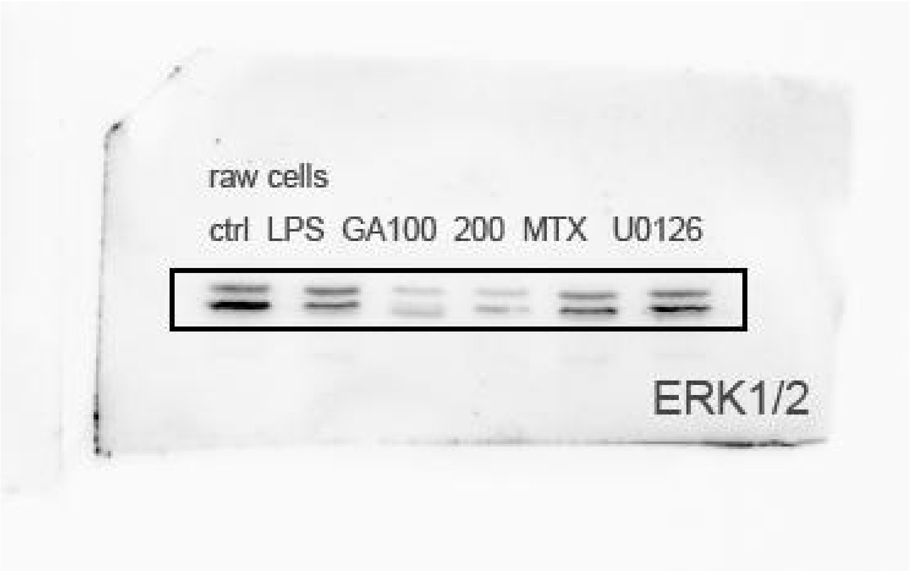

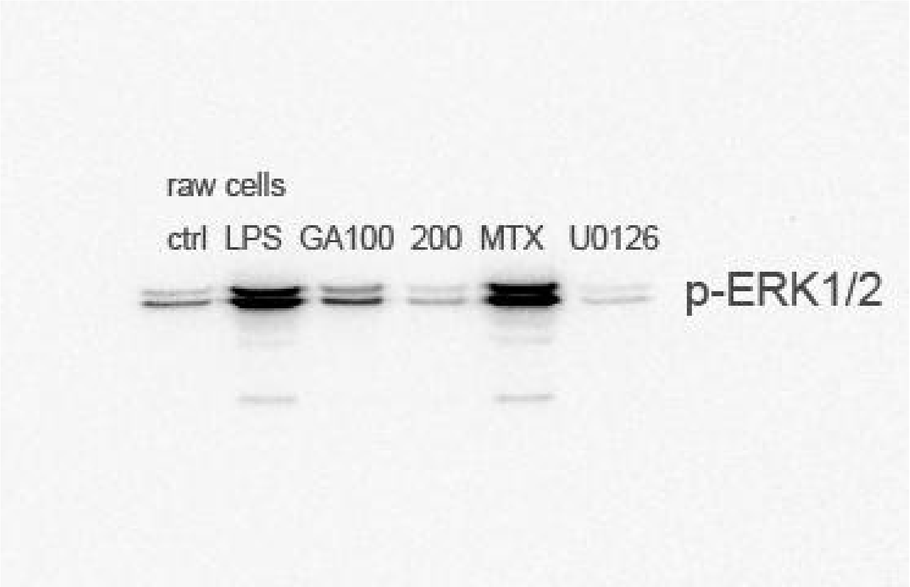

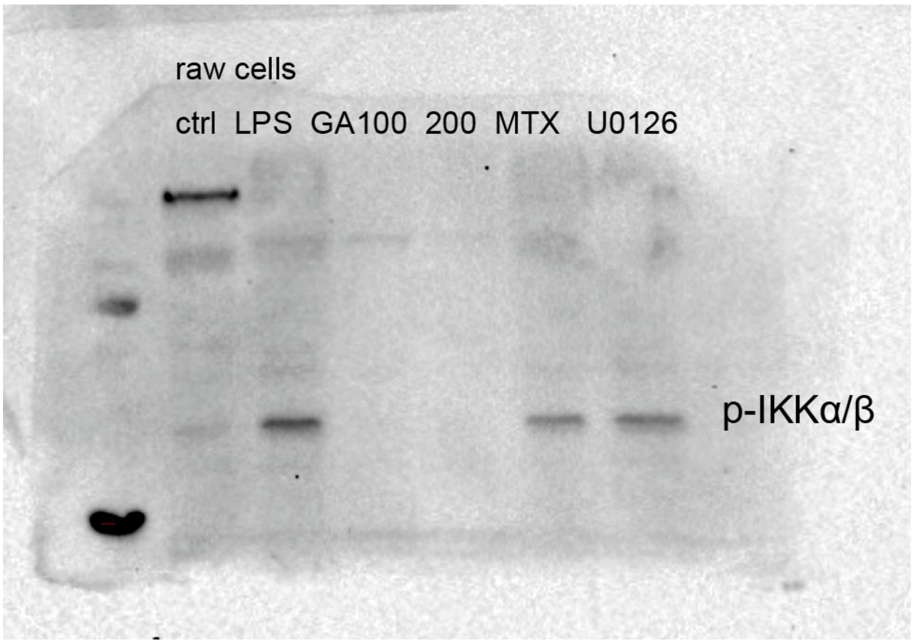

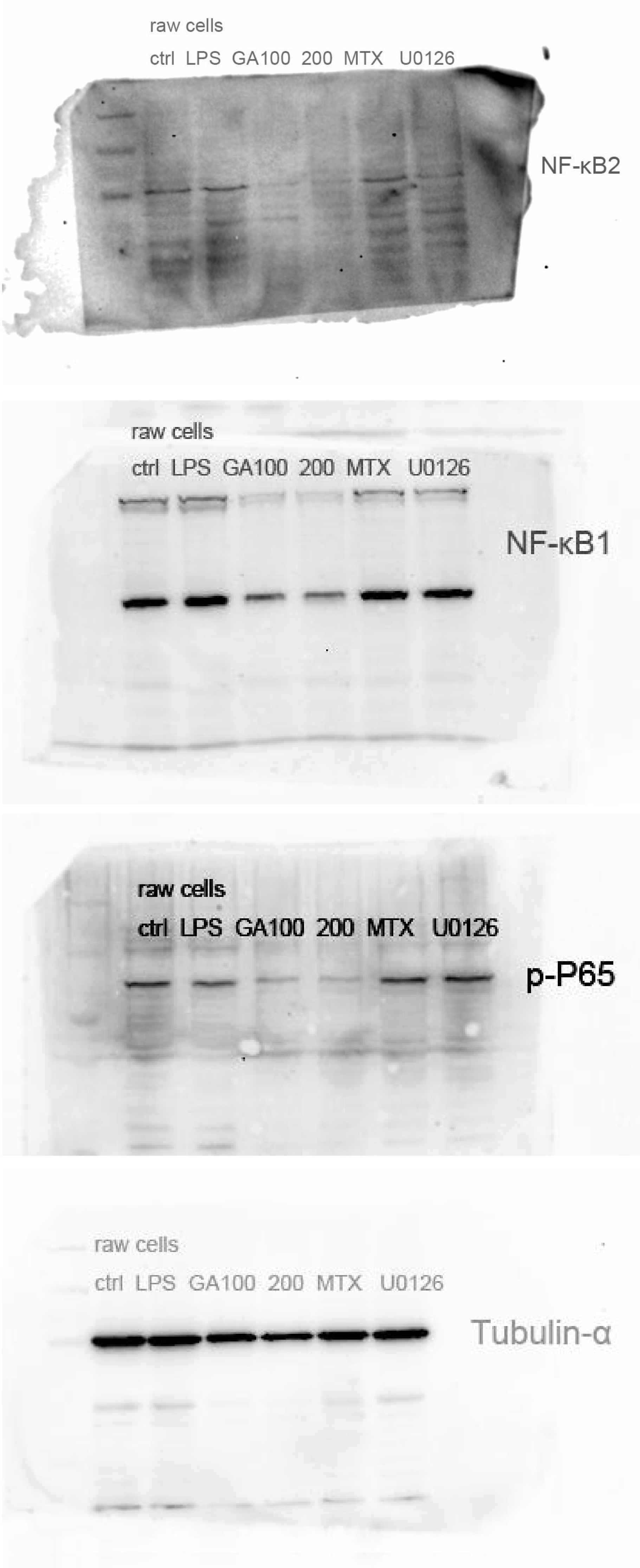
raw cells
ctrl LPS GA100 200 MTX U0126
NF-κB2
raw cells
ctrl LPS GA100 200 MTX U0126
NF-κB1
raw cells
ctrl LPS GA100 200 MTX U0126
p-P65
raw cells
ctrl LPS GA100 200 MTX U0126
Tubulin-α

Second raw WB data:

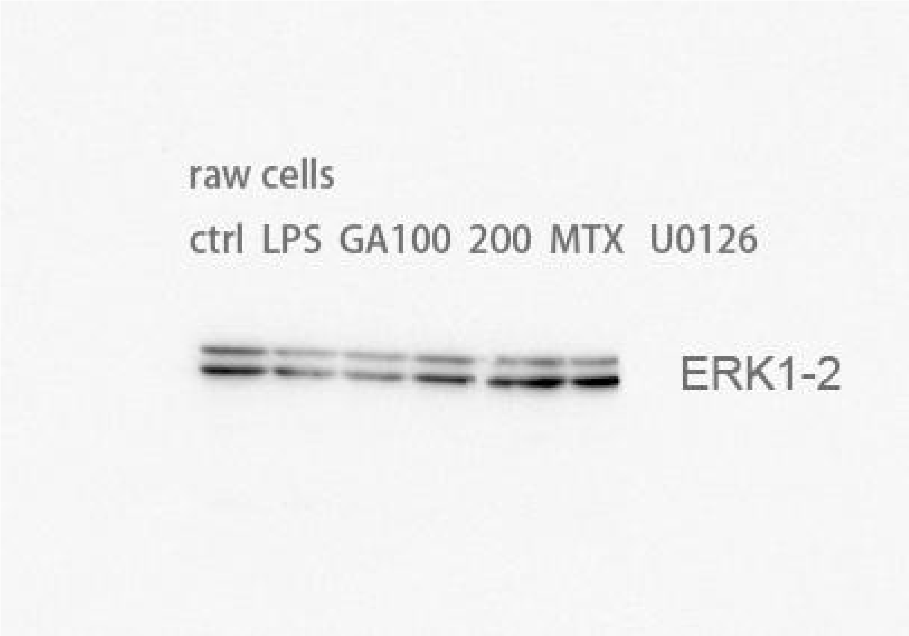

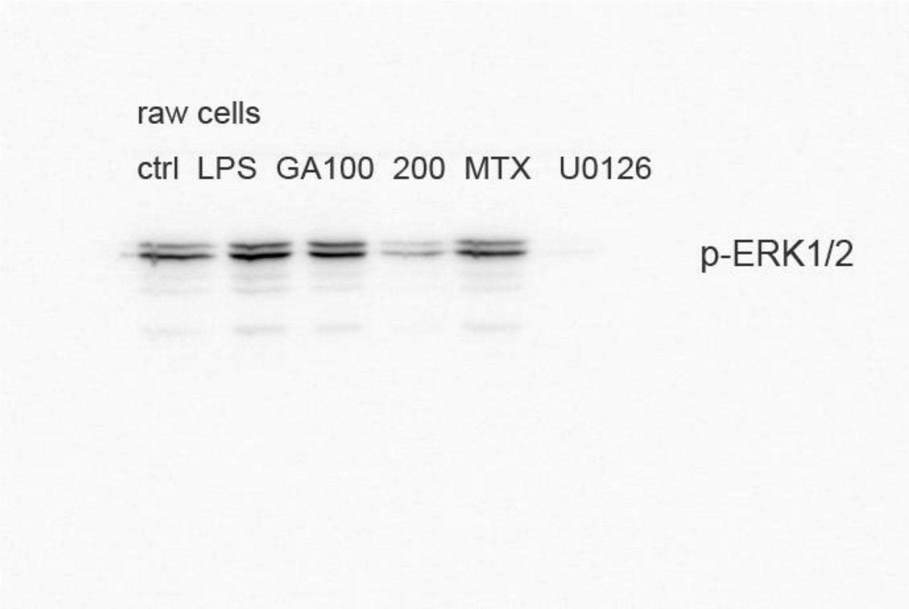

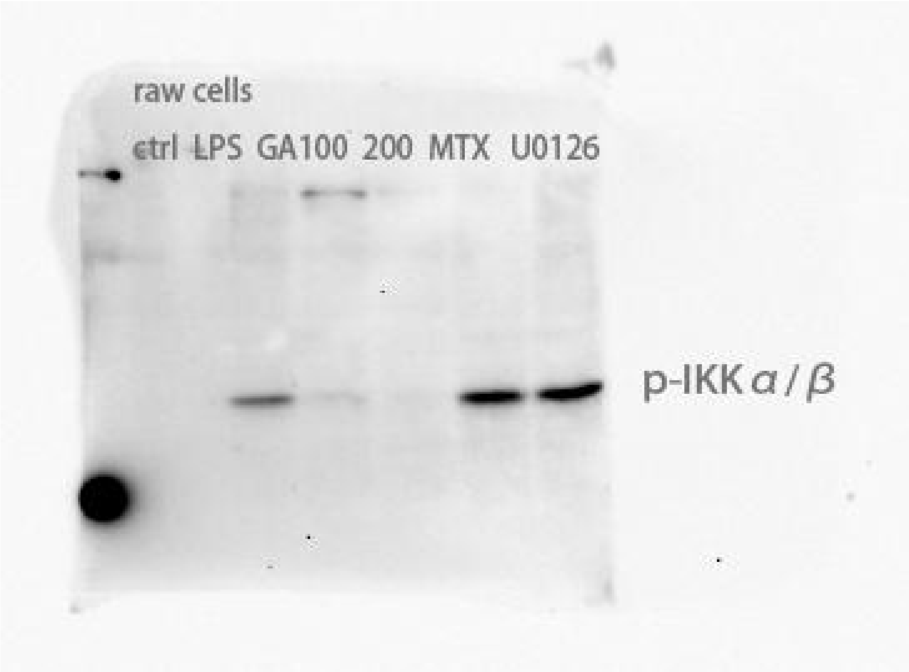

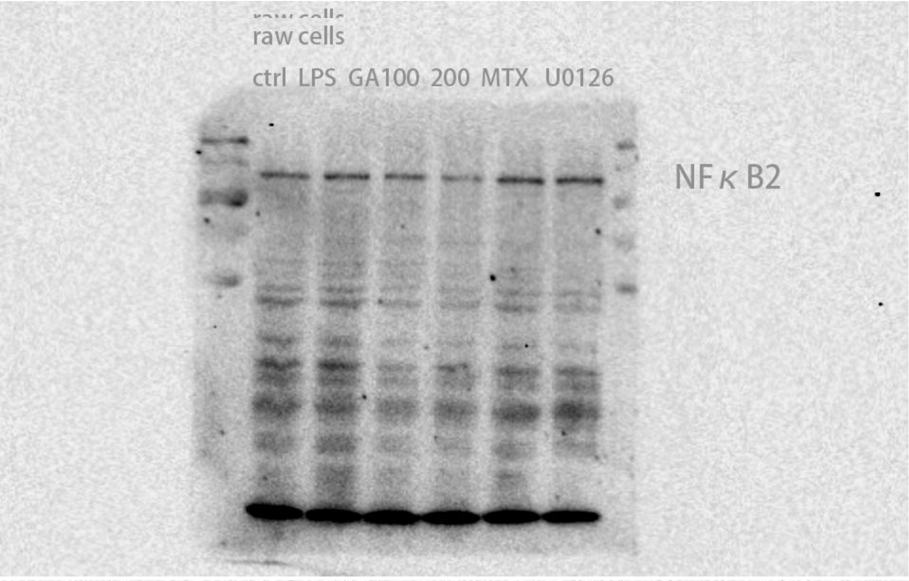
raw cells
ctrl LPS GA100 200 MTX U0126
NFκB2

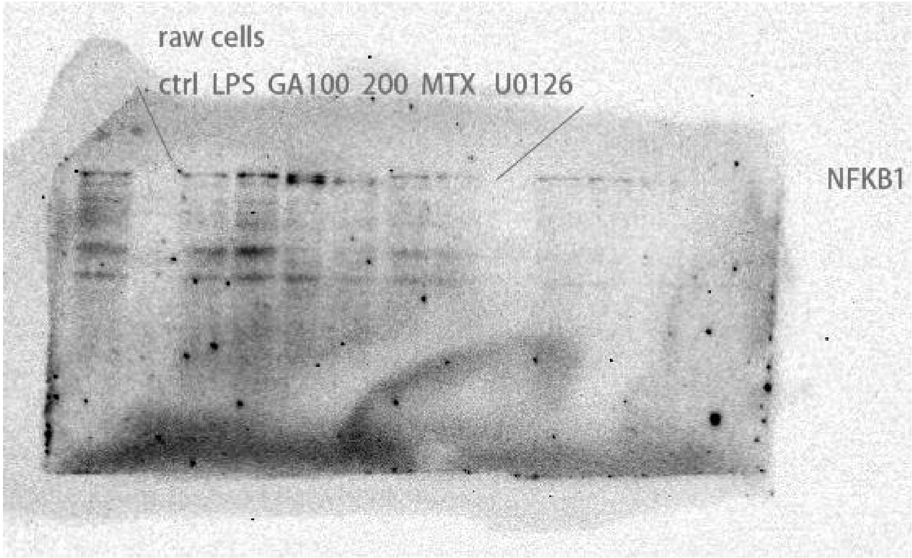
raw cells
ctrl LPS GA100 200 MTX U0126
NFKB1

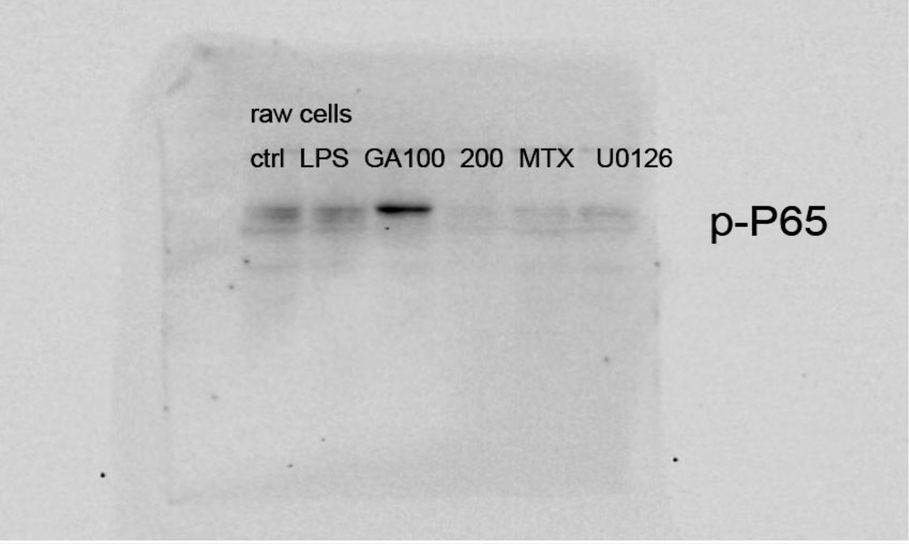
raw cells
ctrl LPS GA100 200 MTX U0126
p-P65

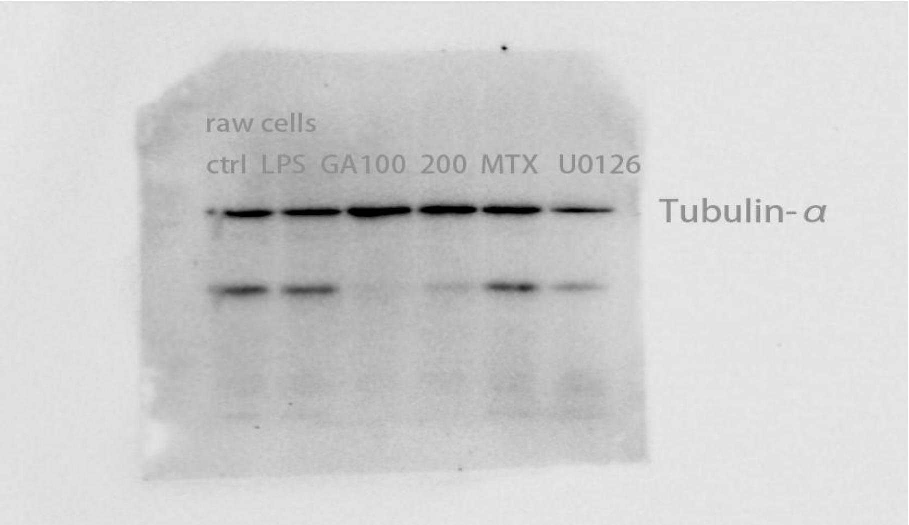
raw cells
ctrl LPS GA100 200 MTX U0126
Tubulin-α

Third raw WB data:

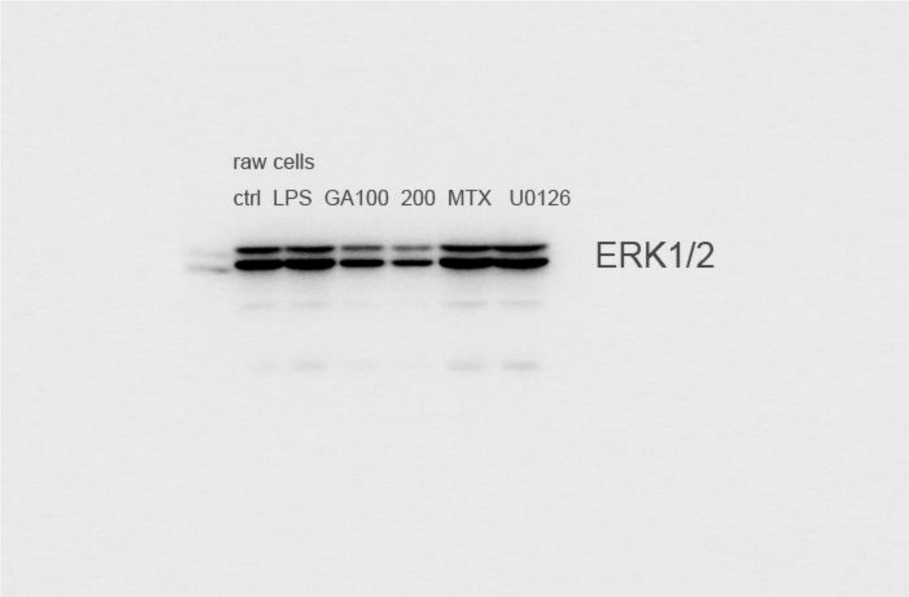

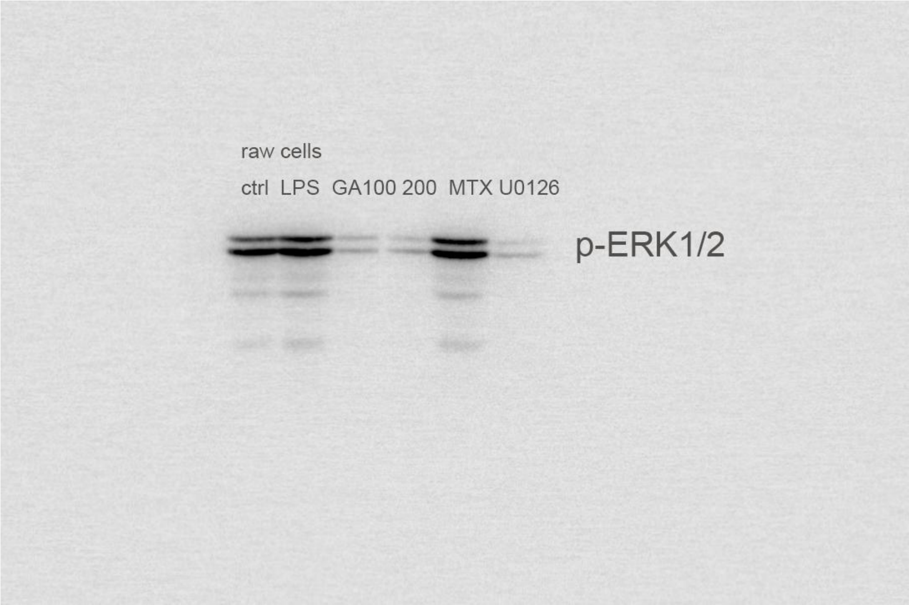

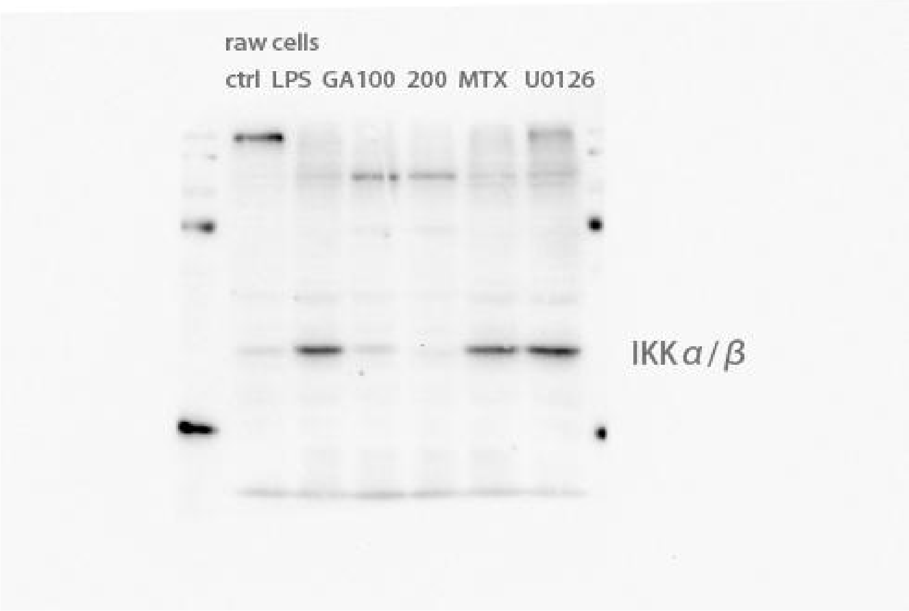

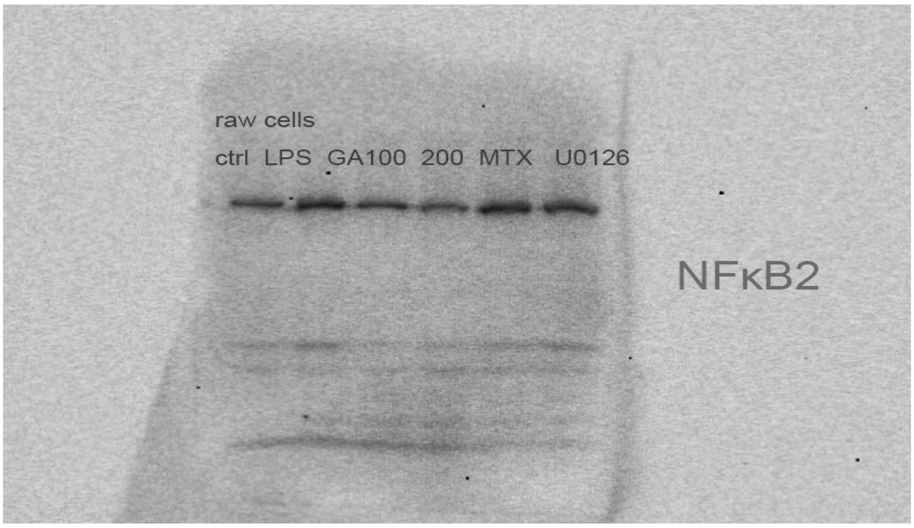
raw cells
ctrl LPS GA100 200 MTX U0126
NFκB2

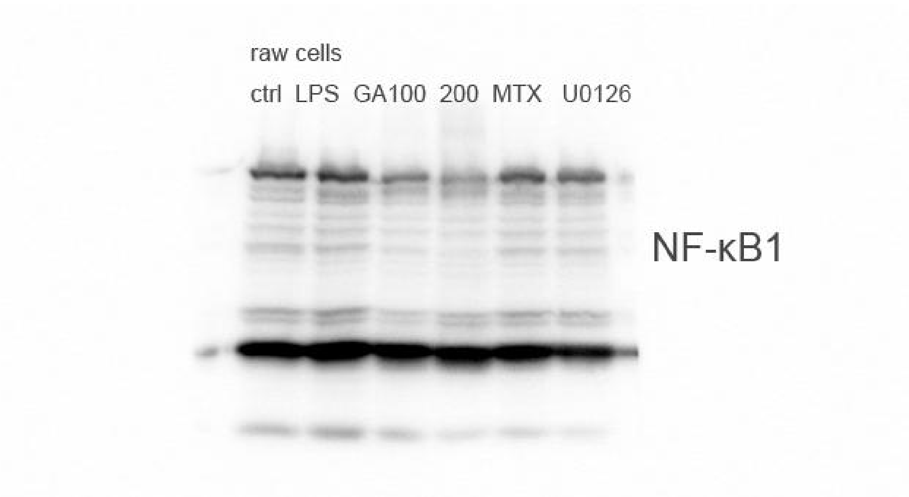
raw cells
ctrl LPS GA100 200 MTX U0126
NF-κB1

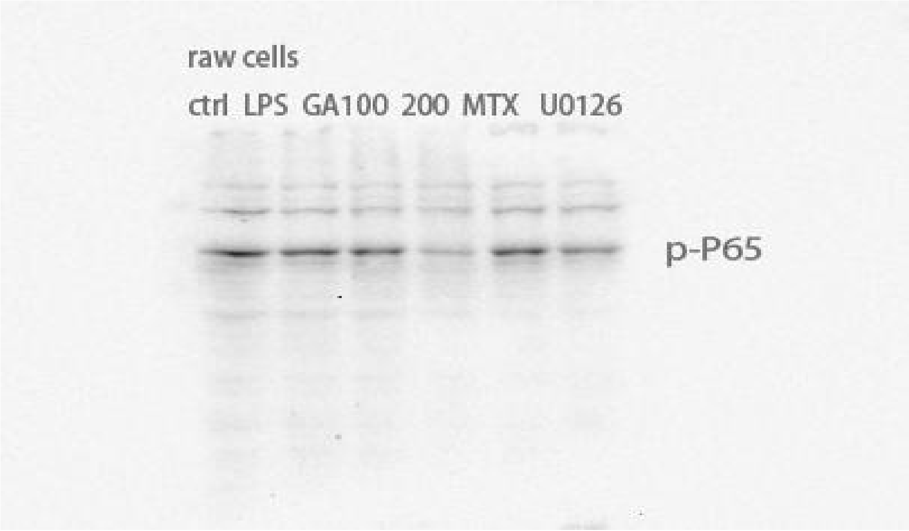
raw cells
ctrl LPS GA100 200 MTX U0126
p-P65

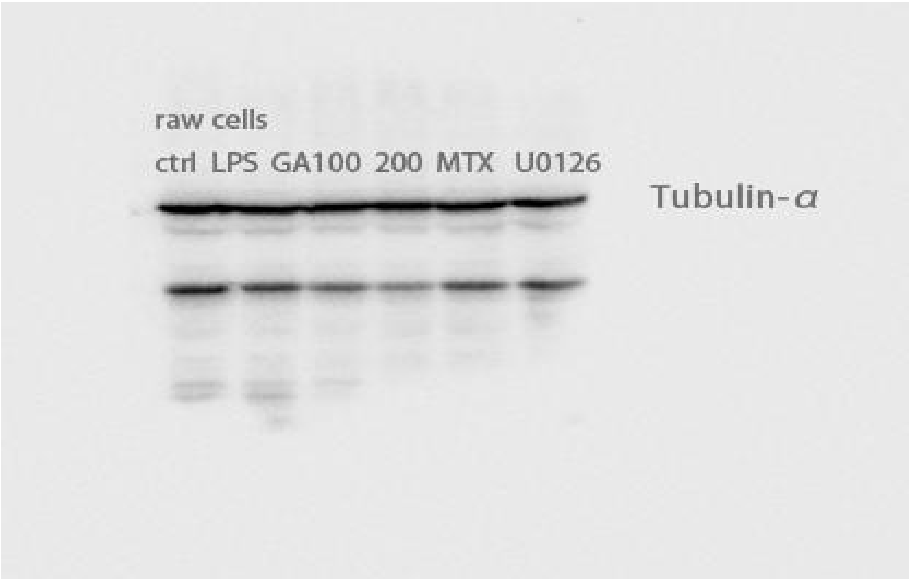
raw cells
ctrl LPS GA100 200 MTX U0126
Tubulin-α
