# Supplementary material for: Anti-inflammatory and Pro-apoptotic Effects of 18beta-Glycyrrhetinic Acid In Vitro and In Vivo Models of Rheumatoid Arthritis
Source: Front Pharmacol. 2021 Jul 26;12:681525. doi: 10.3389/fphar.2021.681525 (PMC8351798; doi:10.3389/fphar.2021.681525)
Supplement: Supplementary file 3 [file DataSheet1.PDF]

广东省中医院实验动物伦理委员会对动物实验研究的审查结果

一、实验项目基本情况：

实验项目名称	基于 COX-2/TxA2 通路介导的炎症微环境研究甘草与甲氨喋呤“相使”与“相畏”关系的减毒增效分子机制	编号	2019024
动物来源	具有生产资质的实验动物生产单位	品种品系	DBA/1 小鼠、基因敲除 DBA/1 小鼠
申请单位	广东省中医院		
联系人	吴晓东	联系电话	18819492432
实验目的	为基于甘草活性成分的类风湿关节炎中西医结合治疗方案开发提供一定的临床前研究证据。		

二、伦理委员会讨论内容：

参加动物实验研究者资格：	1、符合条件 ✓	2、不符合条件	
提供的动物伦理审查材料：	1、符合条件 ✓	2、不符合条件	
动物实验的必要性：	1、必要 ✓	2、不必要	
是否符合动物福利原则：	1、是 ✓	2、否	
动物实验的环境条件是否符合国家标准：	1、是 ✓	2、否	
实验方案：	1、合理 ✓	2、基本合理	3、不合理

三、伦理委员会审议情况：

应到会委员数：5 人	实际到会委员数：5 人	未到委员及原因：
会议地点： 大学城科学院 418 会议室		

四、审议结论：

同意实验

广东省中医院广东省中医药科学院 实验动物伦理委员会

委员	签名
曾 星	
丘小惠	[signature]
郭建文	
郑广娟	[signature]
郑起帆	[signature]
韩凌	
孙景波	[signature]
余谊君	
邓时贵	[signature]
郭世宁	

主任或副主任签名	[signature]
